# Immediate Neuroplastic Changes in the Cortex After iTBS on the Cerebellum of Stroke Patients: A Preliminary fNIRS Study

**DOI:** 10.1155/np/1362222

**Published:** 2025-06-06

**Authors:** Shuo Xu, Shaofan Chen, Ningling Chen, Zhengcong Zhang, Chenfang Liang, Hongwei Huang, Huijie Zou, Haoqing Jiang

**Affiliations:** ^1^Department of Rehabilitation Medicine, Zhangzhou Affiliated Hospital of Fujian Medical University, Zhangzhou, Fujian, China; ^2^Department of Radiology, Zhangzhou Affiliated Hospital of Fujian Medical University, Zhangzhou, Fujian, China

**Keywords:** cortical activation, functional connectivity, intermittent theta-burst stimulation, local spontaneous activity, stroke

## Abstract

**Background and Purpose:** Intermittent theta-burst stimulation (iTBS) targeting the cerebellum represents a promising therapeutic approach, demonstrating efficacy in the rehabilitation of motor and cognitive impairments after stroke. This study aims to evaluate the real-time and immediate effects of cerebellar iTBS on the cerebral cortex of stroke patients.

**Methods:** This study was conducted in a crossover design, initiating with sham-iTBS followed by iTBS after a 24-h washout period. The functional near-infrared spectroscopy (fNIRS) was applied to observe cortical activation from cerebellar iTBS in stroke patients and changes in resting-state functional connectivity (FC) and amplitude of low-frequency fluctuations (ALFF) poststimulation.

**Results:** Compared to sham stimulation, significant enhancement of cortical activation was observed in the left dorsolateral prefrontal cortex (DLPFC; Channel 26, *t* = 2.47, *p*=0.036, Cohen's *d* = 0.783) and left primary motor cortex (PMC; Channel 61, *t* = 2.88, *p*=0.018, Cohen's *d* = 0.907; Channel 62, *t* = 2.62, *p*=0.028, Cohen's *d* = 0.826). Compared to the resting period after sham-iTBS, the resting period following iTBS demonstrated significantly enhanced FC between the temporal cortex (TC) and the somatosensory cortex (SSC) (*p*=0.029), as well as between the frontal eye field (FEF) and the PMC (*p*=0.031). Additionally, the ALFF value of the medial superior frontal gyrus (SFGmed) also increased significantly during the resting period after iTBS (Channel 20, *t* = 5.79, *p*=0.027, Cohen's *d* = 0.63).

**Conclusion:** The application of iTBS to the cerebellum significantly enhances the activation of cognitive and motor areas in the cerebral cortex. Additionally, improved FC between brain regions and increased spontaneous neuronal activity were observed following stimulation. These findings reveal the potential mechanisms by which cerebellar iTBS may facilitate functional recovery in stroke patients.

## 1. Background

Transcranial magnetic stimulation (TMS) has been widely utilized for functional rehabilitation after stroke [1]. The intermittent theta-burst stimulation (iTBS) is a special mode of TMS, offers advantages such as short duration, low intensity, and strong effects [2, 3]. Research indicates that iTBS effectively promotes neural plasticity and improves motor function in stroke patients. A systematic review and meta-analysis confirmed that iTBS enhances motor cortex excitability, thereby improving motor function [4]. Additionally, iTBS has shown potential in improving cognitive functions. For instance, in healthy individuals, Jiang et al. [5] demonstrated that iTBS can enhance cognitive functions like working memory and attention. The application of iTBS in cerebellar studies has also yielded positive results [6, 7].

The cerebellum is primarily involved in motor control and its role in cognition, emotional processing, and motor function rehabilitation has gained increasing attention in recent years [8]. iTBS improves gait and balance by promoting cerebellar cortical plasticity. For example, Zhu et al. [9] showed that cerebellar iTBS combined with conventional physical therapy effectively improves balance and motor function in poststroke hemiplegic patients in the short term. Stafford et al. [10] emphasized that stimulating the cerebellum may induce changes in the cortico–cerebellar pathway, thereby improving motor coordination and functional ability.

Current research focuses primarily on the long-term effects of cerebellar stimulation on stroke recovery and related neuroimaging results. While this approach is important, it leaves a critical gap in understanding the immediate neural changes that occur in the cerebral cortex during and after iTBS application to the cerebellum. The transience of these immediate changes is crucial as it may provide insights into the underlying mechanisms of neural plasticity, which can be targeted in an acute rehabilitation setting. Additionally, the lack of understanding of immediate neural plasticity changes may hinder clinicians' ability to fully comprehend the mechanism of cerebellar iTBS, affecting the optimization and individualization of treatment plans.

This study aims to fill this research gap by synchronously observing immediate neural plasticity changes in the cerebral cortex during cerebellar iTBS using functional near-infrared spectroscopy (fNIRS) equipment. fNIRS is an emerging neuroimaging tool with advantages such as noninvasiveness and real-time monitoring [11]. It has shown promising applications in studying task-based brain activation and resting-state functional connectivity (FC) [12]. Task-based brain activation captures stimulus-evoked hemodynamic changes, while the amplitude of low-frequency fluctuations (ALFFs) measures intrinsic neural oscillations during rest. In addition, FC in the resting state after stimulation can be used to observe the after-effects of iTBS and assess whether it had a continuous influence on the FC patterns of the cortex.

In this study, we investigated the direct effects of cerebellar iTBS on brain activation, FC, and ALFF metrics in stroke patients by fNIRS, providing a new perspective to understand the neuromodulatory mechanisms of iTBS. We hypothesize that cerebellar-targeted iTBS will significantly increase cortical activation levels in stroke patients, enhance FC between related motor areas in the resting state, and augment the low-frequency amplitude of motor control-related cortical regions.

## 2. Methods

### 2.1. Participants

Due to the exploratory nature of this study, which employs a crossover design to investigate changes in fNIRS following a single iTBS intervention, there was no prior effect size data available for a formal sample size calculation. Based on similar studies [13–15] with small sample sizes in this field, our aim was to recruit 10 participants for this preliminary investigation.

A total of 25 stroke patients were initially screened at Affiliated Zhangzhou Municipal Hospital. After applying the following inclusion criteria: (1) Unilateral first stroke was diagnosed by CT or MRI; (2) the age ranged from 40 to 70 years; (3) no comorbid neurological or psychiatric diseases; (4) normal cognition and communication skills; (5) signed informed consent. Ten participants were enrolled. Exclusion reasons included bilateral lesions (*n* = 3), cognitive or language impairment (*n* = 5), comorbidities (*n* = 5), and declined participation (*n* = 2). This study has been approved by the ethics committee of Zhangzhou Hospital of Fujian Medical University (Approval Number: 2024KYZ0348) and registered on the Chinese Clinical Trial Registry.

### 2.2. Study Design

**Figure 1:**
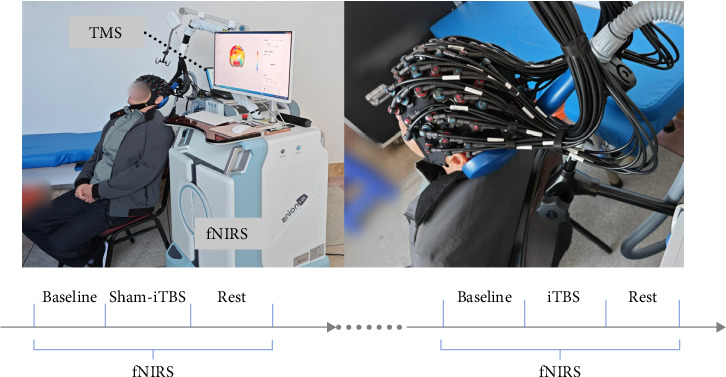
The scene of iTBS intervention and fNIRS signal acquisition procedures.

This study adopted a prospective within-subject crossover design. fNIRS data were synchronously acquired during three phases: (1) a 3-min pre-iTBS baseline recording, (2) real or sham-iTBS stimulation with concurrent fNIRS monitoring, and (3) a 3-min post-iTBS rest period. Each participant received both sham and real-iTBS interventions in a counterbalanced order, separated by a 24-h washout period to eliminate carryover effects [16]. The experimental timeline is illustrated in Figure 1.

### 2.3. iTBS Protocol

iTBS was performed using the Yiruide CCY-1 magnetic stimulator (YRD CCY-1) and a dedicated coil for cerebellar stimulation. We determined the resting motor threshold (RMT) for each subject as follows: (1) Electromyographic activity of the abductor pollicis brevis muscle on the unimpaired side was recorded using surface electrodes. (2) The “hot spot” was identified within the primary motor cortex (PMC) using single-pulse TMS [17]. (3) Starting at a low intensity, single-pulse TMS was administered at the “hot spot” while gradually increasing the intensity until five out of 10 trials produced amplitudes exceeding 50 mV; this intensity was then defined as the individual's RMT [18]. The stimulation target within the cerebellum was determined based on each patient's magnetic resonance T1-weighted structural images and anatomical bony landmarks of the skull [19]. The parameters for iTBS were as follows: intensity set at 80% of the RMT, with three pulses administered at a frequency of 50 Hz within each burst and bursts occurring at a frequency of 5 Hz for 2 s followed by an 8-s interval; a total of 600 pulses were delivered per session [20].

### 2.4. fNIRS Data Acquisition and Processing

Data acquisition was performed using the BrainScope-3000L fNIRS equipment (Wuhan Znion Technology Co., Wuhan, China), which had 106 acquisition channels with a 3-cm distance between channels, covering regions of temporal cortex (TC; Channels 48, 69, 59, 80, 89, 68, 79, and 58), somatosensory cortex (SSC; Channels 60, 70, 71, 81, 82, 87, 88, 77, 78, and 67), premotor cortex and supplementary motor cortex (PreM and SMC; Channels 38, 39, 40, 50, 52, 62, 63, 51, 49, 57, 64, 65, 54, 55, 56, 45, 46, and 47), PMC (Channels 61, 72, 83, 84, 85, 86, 76, and 66), dorsolateral prefrontal cortex (DLPFC; Channels 5, 6, 12, 19, 26, 27, 32, 36, 28, 29, 21, 15, 10, and 11), frontal pole (FPA; Channels 1, 2, 7, 13, 14, 9, 3, and 4), occipital cortex (OC; Channels 103, 104, 91, 90, 94, 95, 99, 100, 101, 102, 97, 98, 93, 92, 105, and 106), Broca (Channels 31, 16, 17, 18, 25, 30, 22, 23, 24, and 37), and frontal eye field (FEF; Channels 33, 41, 42, 43, 44, and 35). Data preprocessing and analysis were conducted using Homer 2 and NirMaster software based on Matlab 2019b. Data preprocessing consists of five steps: including eliminating time intervals irrelevant to the experiment, removing nonexperimental artifacts, converting light intensity to optical density (OD), applying a 0.01–0.1 Hz band-pass filter to eliminate noise and interference, and converting OD to oxygenated hemoglobin concentration based on Beer–Lambert's law [21].

The baseline period represents the resting state collected before stimulation, which is used to calculate the baseline average. The iTBS protocol involved 20 repetitions of 2-s pulses, with 8-s intervals between them, resulting in a 200-s stimulation block. Despite its continuous duration, this block consisted of distinct, temporally spaced events. To model the task, we employed a general linear model (GLM), in which the onset times of each 2-s stimulus were convolved with a canonical hemodynamic response function (HRF). This convolution step captures the typical 4–6 s hemodynamic delay and shape of the fNIRS signal, ensuring temporal alignment between the modeled predictor and the observed physiological response to account for the delayed fNIRS signal [22, 23]. We estimated a single 200-s block *β* value to represent the overall activation, which was then contrasted with the prestimulation baseline. The difference between the two tasks and their respective baseline averages was defined as their individual levels of brain activation [24]. FC analysis was performed by calculating the Pearson correlation coefficient of the time series of oxygenated hemoglobin concentration between channel pairs, defining this coefficient as the FC strength of the corresponding channel pair [25]. Additionally, the ALFF value was calculated by first detrending the original time series of each channel, then, converting it to the frequency domain to extract the frequency band of 0.01–0.1 Hz, and finally calculating the variance of the original signal within this frequency band as the ALFF value [26].

### 2.5. Statistic Analysis

Statistical analysis in this study was performed using R Studio software (version 4.2). Demographic data were expressed as medians and interquartile ranges to reflect the central trend and dispersion of the sample. For brain functional imaging results under the two stimulation conditions, including activation intensity, FC, and ALFF, one-sample *t*-tests (comparing *β* differences with baseline averages), and paired *t*-tests were used for comparisons to evaluate differences between iTBS and sham stimulation. All statistical tests were set at a significance level of *α* = 0.05. In the calculation of ALFF values, we first examined the distribution of the raw data and performed necessary normality tests. If the data violated the normality assumption, we employed non-parametric methods like the Wilcoxon signed-rank test for statistical comparisons. FC strength, represented by Pearson's correlation coefficient (Pearson's *r*), was subjected to Fisher's *z*-transformation. The transformed data were then analyzed using paired-sample *t*-tests to determine the differences between iTBS and sham-iTBS conditions [27]. Additionally, to control for false positive results arising from multiple comparisons, the false discovery rate (FDR) correction method was applied to adjust *p*-values [28].

## 3. Results

### 3.1. Demographics

This study enrolled a total of 25 stroke patients, among which 10 patients met the inclusion criteria. The participants' characteristics are as follows: the gender distribution was nine males and one female, with a median age of 58.5 years (range 42–62 years). All patients had right cerebral cortical or subcortical damage. The median duration of the disease was 3 months. The specific upper limb functional motor assessment (Fugl–Meyer Assessment, upper limb FMA score) had a median of 11.5 points and the lower limb functional motor assessment (lower limb FMA score) had a median of 20.5 points. More personal details for the patients who underwent the intervention are presented in Table 1.

### 3.2. Cortical Activation

**Figure 2:**
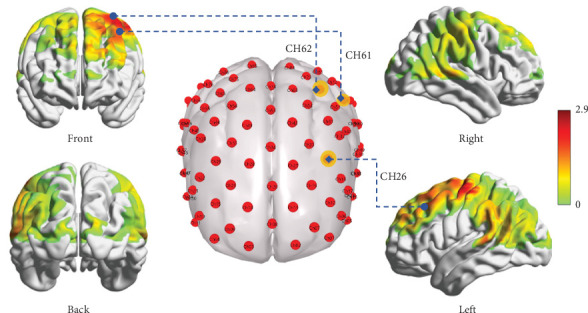
Differences in brain region activation in task states during iTBS versus sham stimulation. CH, channel, CH26 is located in the left dorsolateral prefrontal cortex, and CH61 and CH62 are located in the left primary motor cortex.

By the GLM analysis, we found that during iTBS stimulation, cortical activation in Channels 26, 61, and 62 was significantly higher than in the sham stimulation group (*p* < 0.05; Table 2). Specifically, these channels exhibited stronger oxyhemoglobin signals after real stimulation, indicating a significant increase in neural activity (Figure 2). This result suggests that iTBS stimulation may effectively enhance activation levels in specific brain regions.

### 3.3. FC

**Figure 3:**
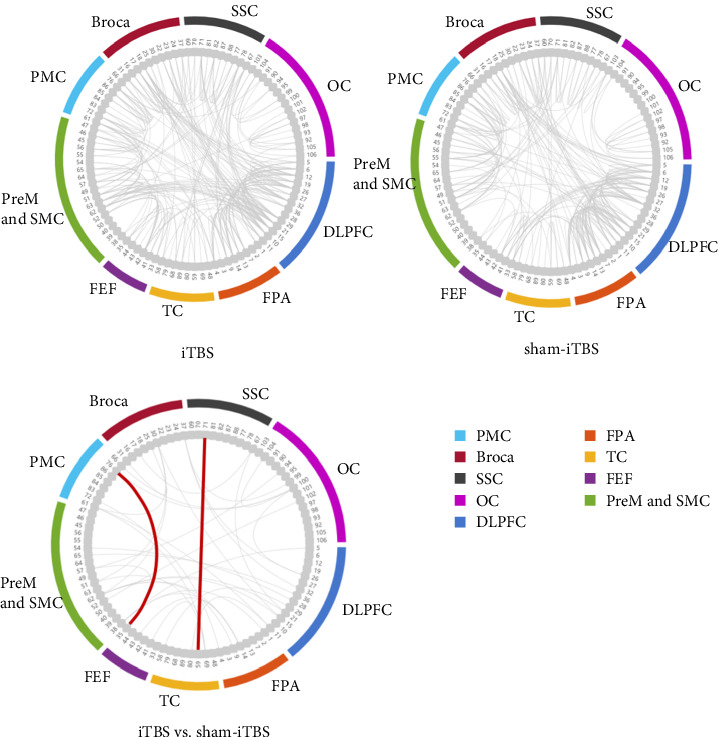
Resting-state brain functional connectivity and differences after iTBS and sham stimulation. DLPFC, dorsolateral prefrontal cortex; FEF, frontal eye field; FPA, frontal pole; OC, occipital cortex; PMC, primary motor cortex; PreM, premotor cortex; SMC, supplementary motor cortex; SSC, somatosensory cortex; TC, temporal cortex.

FC analysis demonstrated significant differences between the resting states after iTBS and sham stimulation. After real stimulation, the strength of FC between multiple channels increased significantly (*p*  < 0.01). Compared to sham stimulation, iTBS significantly enhanced resting-state FC between the TC and SSC (*r* = 0.537, *p*=0.029) and between the FEF and PMC (*r* = 0.321, *p*=0.031), indicating that iTBS applied to the cerebellum promotes connectivity between different regions of the cerebral cortex (Figure 3).

### 3.4. ALFF Analysis

**Figure 4:**
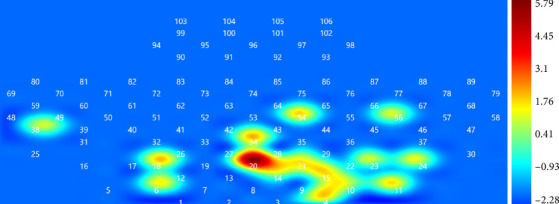
Difference in ALFF values of rest states between post-iTBS and post-sham-iTBS. Channel 20 showed a significant increase in ALFF value in post-iTBS state.

The results of ALFF analysis showed that compared to sham stimulation, after iTBS in the cerebellum, spontaneous neural activity in the brain regions covered by Channel 20 was significantly enhanced (*t* = 5.79, *p*=0.027, FDR correction, Cohen's *d* = 0.63; Table 2). It suggests that iTBS stimulation may positively impact the spontaneous neural activity of this brain region, further supporting the potential of iTBS in improving brain function (Figure 4).

## 4. Discussion

This study investigated the real-time and immediate effects of iTBS on stroke patients, revealing significant improvements in cortical activation, FC, and spontaneous neural activity. Specifically, during iTBS, we observed notably enhanced activation in fNIRS Channels 26, 61, and 62 (with MNI coordinates of [−34, 38, 46, −51, −16, 58] and [−32, −8, 70], respectively). Channel 26 is located in the left DLPFC, while Channels 61 and 62 are in the PMC of the precentral gyrus, brain regions closely associated with cognition and motor execution functions [29, 30]. Compared to the resting state after sham stimulation, the resting state after iTBS showed more functionally connected channels and the region of SFGmed demonstrated higher ALFF values. These findings provide crucial insights into the underlying mechanisms of cerebellar magnetic stimulation in stroke recovery and align interestingly with previous neuroimaging studies on cerebellar TMS [31, 32].

The cerebellum plays a vital role in motor control, coordination, and learning and its modulation of the cerebral cortex has been confirmed by numerous neuroimaging studies [33, 34]. Previous research indicates that the cerebellum influences activity patterns in the cerebral cortex, particularly the prefrontal and motor cortices, through established connections [4, 35]. Zeng et al. [36] found that TMS on the cerebellar can promote the recovery of limb motor dysfunctions after a stroke by enhancing activity in the precentral gyrus, thalamus, and paracentral lobule [37]. Our study results show significantly enhanced activation in the left DLPFC and PMC during cerebellar iTBS in patients with right hemispheric damage, similar to the earlier findings [36, 38]. We believe this cross-hemispheric activation may reflect brain plasticity. When the right hemisphere is damaged, the left hemisphere compensates for the affected areas by enhancing its functions, leading to better motor and cognitive recovery [39].

During stroke recovery, the left hemispheric DLPFC plays a pivotal role. Kim et al. [40] noted that the DLPFC is involved not only in working memory and attention processes but also in motor control. Functional remodeling of the DLPFC can facilitate improvements in cognitive impairments, including enhanced planning and organizational abilities. Therefore, it is a primary target for non-invasive brain stimulation aimed at poststroke cognitive functions [41]. Additionally, enhanced activation of the PMC indicates the potential for motor function recovery in patients. Sohn et al. [42] found that improved motor function after brain stimulation is attributed to increased activity in the PMC. Combined with DLPFC activation, the enhanced co-activation of DLPFC and M1 may provide patients with a more optimized environment for motor learning and execution.

The resting-state analysis revealed more functional connections between channels after iTBS treatment. Compared with post-sham-iTBS, post-iTBS showed significantly enhanced FC between SSC and TC as well as PMC and FEF. This suggests improved neural information transmission between these brain regions. First, the enhanced FC between SSC and TC may reflect an improved efficiency in sensory information processing. Studies have shown that SSC plays a key role in integrating and processing sensory input from the body, while TC is responsible for advanced processing of this information [43]. Therefore, iTBS may have facilitated the transfer of sensory information by promoting synergistic work between the two. The enhanced connectivity between PMC and FEF may be related to the coordination of motor control and visual attention. PMC plays a central role in motor planning and execution, while FEF plays an important role in visual attention and oculomotor control [44]. Thus, iTBS on cerebellar may have facilitated the functional integration between these two regions, thereby improving motor performance and the accuracy of visual responses.

Finally, the results of ALFF in the resting state indicated a significant enhancement in the ALFF values within the SFGmed region following iTBS. This region is recognized as being associated with social cognitive abilities and it also encompasses sensory system coordination, playing a profound role in movement, working memory, cognitive abilities, self-awareness, and emotional regulation [45, 46]. The augmentation of spontaneous neural activity in the SFGmed suggests that iTBS, when applied to the cerebellum, holds therapeutic potential for these functions in stroke patients. Furthermore, as the ALFF values were calculated during the resting state after treatment, they represent, to some extent, the prolonged effects of this therapeutic modality.

Combining previous research has clarified the positive role of cerebellar TMS in stroke rehabilitation [31, 36]. This study provides evidence of real-time and immediate effects for long-term efficacy studies by simultaneously monitoring corticocerebral changes during cerebellar iTBS treatment using fNIRS. Overall, our findings highlight the potential mechanisms of cerebellar-targeted iTBS. Future research can focus on the cortical effect brain regions identified in this study and conduct dual-stimulation target research on the cerebellum and cortex.

## 5. Limitations

First, all participants in this study were patients with right hemispheric damage; therefore, the findings may not be applicable to all stroke patients. Second, due to the exploratory nature of this study, the relatively small sample size may affect the robustness and generality of the results, rendering this analysis exploratory and preliminary. Furthermore, since fNIRS can only observe cortical blood oxygen changes, this study was unable to investigate the effects of iTBS on subcortical structures. Future studies should include patients with left hemispheric damage to observe differences compared to right-sided strokes. It is necessary to increase the sample size and conduct randomized controlled studies to verify the clinical evidence-based effects of iTBS on the cerebellum. Additionally, functional magnetic resonance imaging compatible with magnetic stimulation can be employed to observe the real-time effects on subcortical structures.

## 6. Conclusion

This study demonstrates that iTBS significantly enhances cortical activation, FC, and spontaneous neuronal activity in stroke patients. These findings support the efficacy of iTBS as an intervention that can promote neural remodeling in critical brain regions, offering a promising approach for stroke rehabilitation. Furthermore, this study provides preliminary experimental evidence for future evidence-based research on cerebellar iTBS.

## Figures and Tables

**Table 1:** Demographics and clinical characteristics of the patients.

Patients	Age	TSI	SI	TS	Injury location	FMA-UL	FMA-LL
P1	58	2	S	I	Basal ganglia	10	18
P2	48	4	S	H	Basal ganglia	13	22
P3	60	3	S	I	Corona radiata	8	15
P4	59	1	C	I	Frontoparietal cortex	11	19
P5	52	5	C	I	Parietal cortex	15	25
P6	42	6	C	I	Parietal cortex	9	10
P7	62	2	C	I	Frontoparietal cortex	20	30
P8	59	3	S + C	H	Basal ganglia	12	22
P9	45	4	C	I	Parietal cortex	7	16
P10	61	1	S + C	I	Basal ganglia, corona radiata, frontal cortex	18	31

*Note*: FMA-LL, lower limb score of the Fugl–Meyer assessment; FMA-UL, upper limb score of the Fugl–Meyer assessment.

Abbreviations: SI, site of injury; TS, type of stroke; TSI, time since injury.

**Table 2:** Cortical activation and ALFF between iTBS (post-iTBS) and sham iTBS (post-sham-iTBS).

Brain region (AAL)	Channel	MNI coordinates	*T* value	*p* Value
*β* Values				
DLPFC	26	−34, 38, 46	2.47	0.036
PMC	61	−51, −16, 58	2.88	0.018
PMC	62	−32, −8, 70	2.62	0.028
ALFF				
SFGmed	20	0, 56, 40	5.79	0.027

Abbreviations: DLPFC, dorsolateral prefrontal cortex; PMC, primary motor cortex; SFGmed, medial superior frontal gyrus.

## Data Availability

The data are available from the corresponding author upon reasonable request.
